# Weight status and obesity-related dietary behaviours among culturally and linguistically diverse (CALD) children in Victoria, Australia

**DOI:** 10.1186/s12887-019-1845-4

**Published:** 2019-12-23

**Authors:** Breanna Scott, Kristy A. Bolton, Claudia Strugnell, Steven Allender, Jennifer Marks

**Affiliations:** Global Obesity Centre (GLOBE), Institute of Health Transformation, School of Health and Social Development, Deakin University, Geelong, Victoria Australia

**Keywords:** Children, Overweight, Obesity, Diet, CALD

## Abstract

**Background:**

In developed economies, obesity prevalence is high within children from some culturally and linguistically diverse (CALD) backgrounds. This study aims to identify whether CALD groups in Victoria, Australia, are at increased risk of childhood overweight and obesity, and obesity-related dietary behaviours; compared to their non-CALD counterparts.

**Methods:**

Objective anthropometric and self-report dietary behavioural data were collected from 2407 Grade 4 and 6 primary school children (aged 9–12 years). Children were categorised into CALD and non-CALD cultural groups according to the Australian Standard Classification of Languages. Overweight/obesity was defined according to the World Health Organization growth reference standards. Obesity-related dietary behaviour categories included excess consumption of takeaway foods, energy-dense, nutrient-poor snacks and sugar sweetened beverages. T-tests and chi-square tests were performed to identify differences in weight status and dietary behaviours between CALD and non-CALD children. Logistic regression analyses examined the relationship between CALD background, weight status and dietary behaviours.

**Results:**

Middle-Eastern children had a higher overweight/obesity prevalence (53.0%) than non-CALD children (36.7%; *p* < 0.001). A higher proportion of Middle-Eastern children had excess consumption of takeaway foods (54.9%), energy-dense, nutrient-poor snacks (36.6%) and sugar sweetened beverages (35.4%) compared to non-CALD children (40.4, 27.0 and 25.0%, respectively; *p* < 0.05). Southeast Asian and African children were 1.58 (95% CI = [1.06, 2.35]) and 1.61 (95% CI = [1.17, 2.21]) times more likely, respectively, to consume takeaway foods at least once per week than non-CALD children.

**Conclusions:**

Disparities in overweight/obesity prevalence and obesity-related dietary behaviours among children in Victoria suggest the need for cultural-specific, tailored prevention and intervention strategies.

## Background

Childhood overweight and obesity is highly prevalent [1] and associated with a number of immediate and long-term adverse health outcomes [2, 3]. In 2014–15, 27.4% of Australian children aged 5–17 years were classified as overweight (20.2%) or obese (7.4%) [4]. Demographic and socioeconomic disparities in the distribution of childhood overweight and obesity exist in developed economies such as Australia [5–8]. Several studies have found that the prevalence of childhood overweight and obesity differs according to ethnic or cultural background, with minority groups often bearing a disproportionate burden [9–17].

Culturally and linguistically diverse (CALD) groups constitute a significant proportion of the Australian population. In 2016, 49% of the population were first (born outside of Australia) or second (Australian-born with at least one overseas-born parent) generation Australian, and 21% spoke a language other than English at home [18]. Previous research has identified that Australian children of Middle-Eastern [7, 9–11, 14], Asian [14], Pacific Islander [7, 9, 10] and European [9–11] backgrounds have an increased risk of overweight and obesity compared to children from English-speaking backgrounds (non-CALD). Most recently, data from the 2015 New South Wales (NSW) Schools Physical Activity and Nutrition Survey (SPANS) found that the prevalence of combined overweight and obesity among Middle-Eastern children (42.9%) was almost double that of non-CALD children (21.8%) [19]. While the sample was representative of children in NSW, the findings may not be generalisable to other regions of Australia due to variations in CALD populations across states [18]. Additionally, the categorisation of children into only four broad cultural backgrounds according to the Australian Standard Classification of Languages (Asian, European, Middle-Eastern and English-speaking) may mask variations in overweight and obesity prevalence within CALD subgroups. To develop a better understating of the cultural disparity in childhood overweight and obesity, additional research with disaggregated CALD groups is required.

Despite the known association between dietary behaviours and weight status [20], research on the dietary behaviours of CALD children is limited. Investigating obesity-related dietary behaviours (e.g. high consumption of energy-dense, nutrient-poor [EDNP] foods) [21] among CALD groups may identify practices contributing to their increased weight status which can be targeted. To our knowledge, SPANS is one of the few Australian studies to report on dietary behaviours of CALD children [19]. The 2015 SPANS found that compared to non-CALD children, the prevalence of consuming takeaway food at least once per week was significantly higher among Asian and Middle-Eastern children, while drinking more than one cup of soft drink per day was higher among Middle-Eastern children [19]. Although data on the consumption of several EDNP snack foods (e.g. fried potato products, confectionary) [21] were collected in SPANS, differences in these behaviours according to CALD background were not reported [22].

This study seeks to address gaps in the literature by examining self-report dietary and measured anthropometry data among primary school children to identify whether CALD groups in Victoria, Australia, are at increased risk of childhood overweight and obesity, and obesity-related dietary behaviours compared to their non-CALD counterparts.

## Methods

### Setting

This study utilised baseline (2014) data of children participating in Healthy Together Victoria and Childhood Obesity Study (HTVCO). HTVCO is a repeat cross-sectional study which aims to examine the effect of Healthy Together Victoria (HTV), a community-based lifestyle initiative of the State Government, on the prevalence of overweight and obesity and associated risk factors among school-aged children [23].

### Participants and recruitment

#### Sampling technique

Random sampling was used to invite three primary schools within each of 26 Local Government Areas across Victoria, Australia which reflected the intervention and comparison HTV communities. Where all invited schools declined to participate, additional schools were invited in groups of three until at least one school per Local Government Area accepted. A total of 147 primary schools were invited to participate, of which 48 consented (school-level response rate 32.7%).

All students in Grade 4 (aged 9–10 years) and Grade 6 (aged 11–12 years) enrolled in participating schools were invited to take part in the study through the distribution of a plain language statement and opt-out consent form. Students were considered to have provided informed consent unless an opt-out form signed by parents or guardians was returned to the school or verbal consent was not confirmed by the student at the time of measurement. Data collection took place during Term 3 (July–September) of 2014. Of the 3235 students invited from consenting schools, 377 opted out of the study, and 301 were absent on the data of data collection; resulting in a total of 2557 participants (79% response rate).

### Data collection

Data collection comprised objective measures of height and weight (3–5 min per participant) and a self-reported questionnaire administered via an electronic tablet (20–30 min to complete). All measurements were conducted during the school day by trained research staff.

#### Height and weight

Students’ measurements were taken behind portable screens while wearing a single layer of light clothing and shoes removed. Height was measured using a portable stadiometer (Charder HM-200P Portstad, Charder Electronic Co Ltd., Taichung City, Taiwan) to the nearest 0.1 cm. Weight was measured using an electronic weight scale (A&D Precision Scale UC-321; A7D Medical, San Jose, CA) to the nearest 0.1 kg. All measurements were taken twice, and a third measurement taken if there was a discrepancy (0.5 cm height; 0.5 kg weight) between the first two measures. Mean height and weight measurements were used to generate Body Mass Index (BMI) z-scores according to the World Health Organization (WHO) international growth reference standards [24]. Overweight/obesity (> + 2SD) was defined according to the BMI z-score for age and sex using the WHO growth reference standards [24].

#### Demographic characteristics

Demographic characteristics including age, gender and language spoken at home were collected through a self-reported questionnaire. Language spoken at home was used as a measure of cultural and linguistic diversity. Students were categorised into cultural groups according to the Australian Standard Classification of Languages (1. non-CALD [English-speaking], and 2. CALD [European, Middle-Eastern, Southern Asian, Southeast Asian, Eastern Asian and African]) [25]. Students with missing or ‘other’ language data (*N* = 150) were excluded from the analyses. School postcode was used as a proxy measure of socioeconomic status using the Australian Bureau of Statistics’ Socioeconomic Index for Areas (SEIFA) Index of Relative Socioeconomic Disadvantage [26]. School postcode was also used to categorise students’ locality (i.e. major city, inner regional or outer regional) according to the Australian Bureau of Statistics’ 2011 Australian Statistical Geography Standard (ASGS) Remoteness Structure [27].

#### Dietary behaviours

Dietary behaviours were measured using the Simple Dietary Questionnaire (SDQ) [Parletta N, Frensham L, Peters J, O'Dea K, Itsiopoulos C: Validation of a simple dietary questionnaire with adolescents in an Australian population, unpublished]. Based on the former Australian Dietary Guidelines [28], items from the SDQ measure consumption of fruit, vegetables, takeaway foods, EDNP snack foods, sugar sweetened beverages (SSBs) and sweetened or plain dairy foods. Participants indicated usual number of vegetable and fruit serves per day on a 15-point scale, with possible responses ranging from ‘*none’* to ‘*more than seven serves per day*’ in half-serve increments. Frequency of consumption of takeaway foods was indicated on an 8-point scale, ranging from ‘*rarely or never*’ to ‘*every meal*’. Consumption of all other items were measured on an 8-point scale, ranging from ‘*rarely or never*’ to ‘*3 times or more per day*’. The SDQ has been validated among Australian school children aged 13 to 16 years and demonstrated moderate test-retest reliability and validity when assessed against a 24-h dietary recall. Vegetable consumption demonstrated test–retest reliability of *r* = 0.76 and validity of *r* = 0.42, while fruit consumption demonstrated test–retest of *r* = 0.73 and validity of *r* = 0.57.

Results from a subset of five questions on the SDQ (frequency of consumption of take-away food, EDNP snacks and SSBs) were used to analyse obesity-related dietary behaviours. Individual dietary behaviours were dichotomised as ‘obesity-related’ or ‘non-obesity related’ according to the Australian Dietary Guidelines [21] and previous studies. Obesity-related dietary behaviours included takeaway food ≥1/week (interpreted as excess takeaway consumption), EDNP snacks ≥2/day (interpreted as excess EDNP snacks) and SSBs ≥1/day (interpreted as excess SSBs). Other consumption frequencies were classified as non-obesity related.

### Data analyses and power calculation

All statistical analyses were carried out using Stata v.14 statistics software (StataCorp, College Station, Texas, USA). For descriptive analyses, proportions or means and standard deviations were calculated. T-tests and chi-square tests were performed to identify differences in weight status and dietary behaviours between CALD subgroups and the non-CALD group. Logistic regression analyses were carried out to examine the relationship between CALD background and weight status and dietary behaviours. Two models were investigated: model 1: unadjusted results; model 2: adjusted for potential confounders including age, gender, socioeconomic status, geographical location and school clustering. Statistical significance was set at *p* < 0.05.

#### Sample size calculation

A sample of 327 participants in each group (CALD and non-CALD, total *N* = 654) was required to detect a 10% difference in overweight/obesity prevalence with 80% power and an alpha level of 0.05. These sample size calculations were based on data from the 2015 SPANS survey whereby a 21.1% difference in overweight/obesity prevalence between Middle-Eastern background and English-speaking primary school children was observed [19].

## Results

### Participant characteristics

Of the 2407 participants, 50.2% were female, and the mean age was 11 years (SD = 1.1). More than half (54.4%) of children lived in major cities, and 56.7% lived in areas categorised as being in the lower two SEIFA quintiles. Just under a third (29.2%) of participants were classified as CALD: 7.9% European, 6.1% Middle-Eastern, 4.9% Southern Asian, 4.1% Southeast Asian, 3.4% African and 2.9% Eastern Asian backgrounds.

### Prevalence of overweight and obesity

Over a third (37.3%) of all participants were classified as overweight or obese (Table 1). Except for Middle-Eastern children, no significant differences in overweight/obesity prevalence were observed between CALD subgroups and the non-CALD group. More than half (53%) of Middle-Eastern children were categorised as overweight/obese, compared to 38.5% of non-CALD children (*p* < 0.001).

**Table 1:** Descriptive statistics of Victorian primary school children by CALD background

Characteristic	Total	non-CALD (reference)	European	*p*	Middle- Eastern	*p*	Southern Asian	*p*	Southeast Asian	*p*	Eastern Asian	*p*	African	*p*
*n* (%)	2407	1703 (70.8)	189 (7.9)		146 (6.1)		119 (4.9)		98 (4.1)		70 (2.9)		82 (3.4)	
Age in years (mean ± SD)	11.0 ± 1.1	11.1 ± 1.1	10.9 ± 1.1	0.097	10.9 ± 1.1	0.054	10.7 ± 1.0	**0.002**	11.1 ± 1.1	0.902	10.9 ± 1.1	0.200	10.7 ± 1.1	**0.008**
Female (%)	50.2	51.1	45.5	0.145	50.7	0.926	44.5	0.167	52.0	0.854	52.9	0.771	45.1	0.291
Locality (%)^a^														
Major city	54.4	41.2	77.3	**<0.001**	97.0	**<0.001**	91.6	**<0.001**	83.7	**<0.001**	87.1	**<0.001**	82.9	**<0.001**
Inner regional	37.5	48.6	16.4	**<0.001**	3.4	**<0.000**	7.6	**<0.001**	8.2	**<0.001**	12.9	**<0.001**	15.9	**<0.001**
Outer regional	8.1	10.2	6.4	0.094	0.0	**<0.001**	0.8	**0.001**	8.2	0.523	0	**0.005**	1.2	**0.008**
SEIFA (%)^b^														
1st quintile (most disadvantaged)	28.7	31.5	29.3	0.548	13.5	**<0.001**	13.6	**<0.001**	41.7	**0.037**	10.3	**<0.001**	19.2	**0.022**
2nd quintile	28.0	27.9	23.8	0.233	29.8	0.637	38.1	**0.018**	35.4	0.113	27.9	0.998	12.8	**0.003**
3rd quintile	21.2	16.7	28.2	**<0.001**	42.6	**<0.001**	29.7	**<0.001**	12.5	0.286	29.4	**0.006**	51.3	**<0.001**
4th quintile	18.9	21.2	16.0	0.101	9.2	**0.001**	13.6	0.047	7.3	**0.001**	25	0.456	10.3	**0.020**
5th quintile (least disadvantaged)	3.3	2.7	2.8	**0.004**	5.0	0.133	5.1	**<0.001**	3.1	**0.006**	7.4	**0.027**	6.4	0.060
BMI-z (mean ± SD)	0.7 ± 1.2	0.7 ± 1.2	0.8 ± 1.3	0.195	0.9 ± 1.4	**0.009**	0.3 ± 1.4	**0.002**	0.4 ± 1.2	0.070	0.5 ± 1.2	0.396	0.4 ± 1.4	**0.044**
Overweight/obese (%)^c^	37.3	36.7	41.5	0.217	53.0	**<0.001**	29.9	0.154	28.7	0.116	34.8	0.740	34.2	0.634
Dietary behaviour (%)														
Takeaway ≥1/week	43.4	40.4	45.4	0.185	54.9	**0.001**	52.1	**0.012**	58.2	**0.001**	35.7	0.437	56.8	**0.003**
EDNP snacks ≥2/day	28.4	27.0	31.6	0.184	36.6	**0.014**	31.1	0.330	30.9	0.395	24.3	0.619	30.9	0.443
SSB ≥1/day	25.4	25.0	25.4	0.911	35.4	**0.007**	18.6	0.120	21.4	0.423	17.1	0.134	36.6	**0.019**

^a^ based on the ABS Australian Statistical Geographical Standard Remoteness Structure; ^b^ based on the ABS SEIFA Index of Relative Socioeconomic Disadvantage; ^c^ according to WHO World Health Organisation classification; *p*-values in bold indicate a statistically significant difference (*p*<0.05) between CALD or CALD subgroup and reference population (non-CALD), determined using t-test or chi-squared test, as appropriate; *BMI* body mass index, *CALD* culturally and linguistically diverse, *EDNP* energy-dense, nutrient-poor, *SD* standard deviation, *SEIFA* Socio-Economic Indexes for Areas, *SSB* sugar sweetened beverage

### Dietary behaviours

Across CALD subgroups, several variations in dietary behaviours were found (Table 1). African, Southeast Asian, Southern Asian and Middle-Eastern children had a significantly higher weekly consumption of takeaway foods compared to non-CALD children (*p* < 0.05). Frequent takeaway food consumption was highest among the Southeast Asian group, with 58.2% of children. Except the Middle-Eastern group, there were no significant differences in the proportion of children consuming two or more sugary or salty snacks per day among CALD subgroups compared to the non-CALD group. Snack consumption was almost 10% higher among Middle-Eastern compared to non-CALD children (36.6 and 27.0%, respectively; *p* = 0.14). SSB consumption frequency was significantly higher among Middle-Eastern (35.4%, *p* = 0.007) and African (36.6%, *p* = 0.019) groups compared to the non-CALD group (25.0%).

Model 1 (unadjusted) shows children of Middle-Eastern backgrounds as 1.94 times more likely than non-CALD children to be overweight or obese (CI = [1.36, 2.76]; *p* < 0.001) (Table 2). This association remained statistically significant after adjustment (OR = 1.99; 95% CI = [1.31, 3.04]; *p* = 0.001) (Model 2). No other significant associations in the odds of overweight and obesity among the other CALD sub-group compared to the non-CALD group were observed.

**Table 2:** Logistic regression analysis examining influence of CALD background on overweight/obesity and dietary behaviours

CALD background/ dietary behaviour	Model 1 (unadjusted)			Model 2 (adjusted)		
	OR	95% CI	*p*	OR	95% CI	*p*
European						
Overweight/obesity	1.22	0.89–1.67	0.218	1.32	0.83–2.11	0.248
Takeaway ≥1/week	1.23	0.91–1.67	0.186	1.11	0.82–1.50	0.519
EDNP snacks ≥2 /day	1.25	0.90–1.73	0.185	1.18	0.85–1.65	0.332
SSB ≥1/day	1.02	0.72–1.45	0.911	0.89	0.64–1.22	0.460
Middle-Eastern						
Overweight/obesity	1.94	1.36–2.76	**< 0.001**	1.99	1.31–3.04	**0.001**
Takeaway ≥1/week	1.80	1.28–2.53	**0.001**	1.54	0.99–2.37	0.053
EDNP snacks ≥2 /day	1.56	1.09–2.22	**0.014**	1.50	1.05–2.15	**0.026**
SSB ≥1/day	1.65	1.15–2.36	**0.007**	1.57	0.85–2.92	0.151
Southern Asian						
Overweight/obesity	0.73	0.48–1.13	0.156	0.71	0.41–1.13	0.213
Takeaway ≥1/week	1.61	1.11–2.33	**0.013**	1.28	0.84–1.96	0.251
EDNP snacks ≥2 /day	1.22	0.82–1.83	0.331	1.14	0.77–1.69	0.513
SSB ≥1/day	0.69	0.43–1.11	0.121	0.62	0.37–1.06	0.080
Southeast Asian						
Overweight/obesity	0.69	0.44–1.10	0.118	0.68	0.43–1.08	0.099
Takeaway ≥1/week	2.06	1.36–3.11	**0.001**	1.58	1.06–2.35	**0.024**
EDNP snacks ≥2 /day	1.21	0.78–1.89	0.396	1.17	0.62–2.20	0.638
SSB ≥1/day	0.82	0.50–1.34	0.423	0.68	0.30–1.55	0.358
Eastern Asian						
Overweight/obesity	0.92	0.55–1.52	0.741	0.94	0.55–1.56	0.808
Takeaway ≥1/week	0.82	0.50–1.35	0.438	0.72	0.42–1.22	0.224
EDNP snacks ≥2 /day	0.87	0.50–1.52	0.619	0.80	0.49–1.32	0.397
SSB ≥1/day	0.62	0.33–1.17	0.137	0.64	0.34–1.19	0.159
African						
Overweight/obesity	0.89	0.56–1.43	0.634	0.93	0.70–1.24	0.622
Takeaway ≥1/week	1.94	1.24–3.05	**0.004**	1.61	1.17–2.21	**0.003**
EDNP snacks ≥2 /day	1.21	0.75–1.96	0.443	1.08	0.81–1.45	0.591
SSB ≥1/day	1.73	1.09–2.75	**0.021**	1.76	1.30–2.38	**< 0.001**

Reference: non-CALD children. Model 2 adjusted for age, gender, SEIFA, locality and school clustering. *p*-values in bold indicate a statistically significant association (*p* < 0.05)

*CALD* culturally and linguistically diverse, *CI* confidence interval, *EDNP* energy-dense nutrient-poor, *OR* odds ratio, *SEIFA* Socioeconomic index for areas, *SSB* sugar sweetened beverage

Associations with dietary behaviours differed across CALD subgroups. Apart from takeaway food and SSB consumption in the Middle-Eastern group, all other associations between CALD subgroups and dietary behaviours remained statistically significant after adjustment. In the adjusted model, children of Southeast Asian and African background were 1.58 (95% CI = [1.06, 2.35]; *p* = 0.024) and 1.61 (95% CI = [1.17, 2.21]; *p* = 0.003) times more likely, respectively, to consume takeaway one or more times per week than non-CALD children. Compared to the non-CALD group, children of Middle-Eastern backgrounds had 1.5 times the odds of consuming two or more EDNP snacks per day (95% CI = [1.05, 2.15]; *p* = 0.026). The odds of consuming one or more SSBs per day were significantly higher among children of African backgrounds (OR = 1.76; 95% CI = [1.30, 2.38]; *p* < 0.001).

## Discussion

This study examined differences in weight status and dietary behaviours among CALD and non-CALD children in Victoria. Overall, rates of overweight and obesity were high among the sample, although children of Middle-Eastern backgrounds were found to have significantly higher odds of overweight/obesity compared to non-CALD children. Poorer dietary behaviours were observed in several CALD subgroups compared to the non-CALD group. A higher proportion of Middle-Eastern children were found to have excess takeaway food, EDNP snack and SSBs consumption compared to non-CALD children. The odds of excess takeaway consumption were significantly higher among Southeast Asian and African children compared to their non-CALD counterparts. African children also had a higher likelihood of excess SSB consumption. These findings collectively present intervention opportunities to improve poor dietary behaviours. For example increasing the promotion of, and access to healthy food options and increasing water consumption using culturally appropriate strategies.

### Overweight and obesity prevalence

In the present study, over half (53%) of children of Middle-Eastern backgrounds were classified as overweight or obese, compared to 36.7% of non-CALD children. This finding is supported by previous Australian research, which has consistently identified high rates of overweight and obesity among Middle-Eastern background children over the past two decades [9, 29]. The proportion of overweight or obese Middle-Eastern background and non-CALD children in the present study is considerably higher than that reported in the 2015 SPANS (37.5 and 13.3%, respectively) [19]. This is likely due to methodological differences in SPANS, such as a opt-in recruitment resulting in a response rate of 67.9% and the use of International Obesity Taskforce cut-points to classify weight status, which typically has more conservative estimates of overweight and obesity than the WHO system [30, 31]. Nevertheless, findings from the present study add to the large body of evidence identifying a need to address the disproportionate prevalence of overweight and obesity among children of Middle-Eastern backgrounds.

Contrary to prior research, overweight and obesity prevalence among all other CALD subgroups did not differ significantly to that of non-CALD children. Australian children of African [9], European [9–11] and Pacific Islander [9, 10] backgrounds have previously been found to have an increased risk of excess weight. Due to the small sample sizes of CALD subgroups in the present study, there may not have been sufficient power to detect differences in weight status compared to the non-CALD group. Differences in identified at-risk groups between the present and previous studies may also be somewhat explained by alternate methods of classifying CALD background. For instance, Waters et al. [11] determined CALD background according to maternal region of birth. An additional 24% of children were classified as CALD using this method compared to language spoken at home, thus resulting in a higher potential of identifying overweight and obesity among those classified as CALD [11]. Additionally, children of Pacific Islander backgrounds were excluded from analysis in the present study due to insufficient numbers. As such, future research should endeavour to recruit a sample large enough to include and be representative of all CALD subgroups so that at-risk groups can be accurately identified.

### Dietary behaviours

Excess takeaway consumption appeared to be a significant issue among several CALD subgroups in the present study. Excluding children of European and Eastern Asian backgrounds, more than half of children among all other CALD subgroups consumed takeaway one or more times per week. This is concerning, as takeaway foods are typically high in energy, saturated fat, salt and sugar, and have been associated with excess weight in children [32–34]. Furthermore, higher consumption of takeaway foods has been shown to not only be associated with an increased risk of obesity; but also other metabolic disorders such as insulin resistance and type 2 diabetes; along with cardiovascular disorders [35]. There are many aspects that may influence takeaway consumption, including its relative affordability, accessibility, convenience and media advertising [36, 37]. However, the persisting relationship between takeaway consumption and Southeast Asian or African background in the adjusted regression model is indicative of an independent cultural influence. For example, high cultural regard for Western foods and dietary acculturation have been identified as factors influencing takeaway consumption among African migrants in Australia [38, 39].

Overall, the proportion of children consuming one or more SSBs per day within CALD subgroups was similar to that of non-CALD group in the present study. However, a significantly higher prevalence of SSB consumption was identified among Middle-Eastern and African background children. Furthermore, compared to non-CALD children, African children had almost two times the odds of excess SSB consumption. Similar results among children of Middle-Eastern backgrounds were reported in SPANS, who were found to have a significantly higher prevalence of soft drink consumption and availability of soft drinks in the home compared with non-CALD children [19]. To our knowledge, this is the first study to investigate SSB consumption among children of African backgrounds in Australia. Considering the association between SSB consumption and increased weight status [40], this behaviour among children of African backgrounds warrants further investigation. Additionally, this finding highlights the importance of investigating obesity-related dietary behaviours among minority CALD subgroups.

Children of Middle-Eastern backgrounds were also found to have a significantly higher prevalence of consuming two or more sugary or salty snacks per day compared to non-CALD children. This finding is consistent with previous research [14, 19, 41]. In an analysis of the cumulative intake of ‘junk’ foods (e.g. sweet or savoury biscuits, ice-cream) among children in NSW, Boylan et al. [41] found that over half of children from Middle-Eastern backgrounds were high junk food consumers. It has also been shown that children of Middle-Eastern backgrounds are often rewarded for good behaviour with sweets [14, 19], which may partially explain the higher odds of excess snack consumption compared to non-CALD children in the present study.

### Strengths and limitations

Data for this study were drawn from a large sample of Victorian primary school children spanning broad demographic and geographic areas. At a school-level, non-response bias was minimised due to the opt-out recruitment process. Therefore, the present study may have more accurately estimated overweight/obesity prevalence compared to research with opt-in procedure [42]. A further strength is the use of standardised protocols for the collection of height and weight measurements.

There are also some limitations of this research to consider. Firstly, as these data are cross-sectional, causality cannot be inferred. In addition, BMI is not able to distinguish between fat and fat-free mass and therefore has limitations as a measure of overweight and obesity at an individual level. However, at a population-level, BMI is an accurate, cost and time efficient measure for population surveillance of childhood overweight and obesity and has very high specificity [43]. Dietary behaviours were self-reported by children and were therefore subject to recall and measurement bias. Food frequency questionnaires are commonly completed by parent proxy in children less than 12 years old due to cognitive development among young children potentially influencing food recall and estimated portion sizes [44]. However, parents of children may not be aware of foods consumed out of the home, and there is research to suggest that children as young as 8 years old are more accurate reporters of food frequency consumption than their parents [45]. The classification of CALD background according to language spoken at home is a potential limitation of this study, as it is possible that some CALD children speak predominantly English at home. Finally, while the sample was large, there was not sufficient power among all CALD subgroups to detect differences in weight status or dietary behaviours compared to non-CALD children. Future research examining differences in CALD groups speaking predominantly English compared to those speaking predominantly their native language would shed light on whether disparities between groups are influenced by lifestyle, genetic or a combination of factors. Examining the effects of acculturation on behaviours that influence weight status in migrant children living in Australia would also be beneficial [14]. In depth qualitative research to understand dietary choices in these CALD groups is also required.

## Conclusion

This study identified a higher prevalence of overweight and obesity among children of Middle-Eastern backgrounds and revealed disparities in dietary behaviours among CALD compared to non-CALD children. These modifiable dietary behaviours present as areas for targeted and culturally appropriate childhood overweight and obesity intervention and prevention strategies, particularly among children of Middle-Eastern backgrounds. Additional research is required to understand the underlying causes that uniquely contribute to obesity-related dietary behaviours among CALD children.

## Supplementary information

**Additional file 1: Table S1.** Descriptive statistics of Victorian primary school children by CALD background.

## Data Availability

The datasets used and/or analysed during the current study are available from the corresponding author on reasonable request.

## Abbreviations

CALD: Culturally and linguistically diverse
EDNDP: Energy-dense, nutrient-poor
HTV: Healthy Together Victoria
HTVCO: Healthy Together Victoria and Childhood Obesity Study
NSW: New South Wales
SDQ: Simple Dietary Questionnaire
SEIFA: Socioeconomic Index for Areas
SPANS: Schools Physical Activity and Nutrition Survey
SSB: Sugar sweetened beverage
WHO: World Health Organization
